# *HIF1α* and *HIF2α* immunoreactivity in epithelial tissue of primary and recurrent pterygium by immunohistochemical analysis

**DOI:** 10.1007/s10792-023-02855-3

**Published:** 2023-09-08

**Authors:** Kristina Joana Schoelles, Katharina Kemper, Gottfried Martin, Daniel Boehringer, Katarzyna Brinks, Hans Mittelviefhaus, Thomas Reinhard, Claudia Auw-Haedrich

**Affiliations:** Eye Center, Medical Center - University of Freiburg, Killianstraße 5, 79106 Freiburg, Germany

**Keywords:** Pterygium, Conjunctiva, *HIF1α*, *HIF2α*, Immunohistochemistry

## Abstract

**Purpose:**

Hypoxia-inducible factors (*HIFs*) are considered to play a significant role in the pathogenesis of pterygium. The aim of this study was to investigate the relative expression or immunoreactivity of *HIF1α* and *HIF2α* in the epithelium of primary pterygium, recurrences and healthy conjunctiva.

**Methods:**

Immunohistochemical staining was performed with antibodies against *HIF1α* and *HIF2α*, respectively, on 55/84 primary pterygium specimens, 6/28 recurrences and 20/20 control tissues (healthy conjunctiva).

**Results:**

Immunohistochemical staining revealed lower epithelial immunoreactivity of *HIF1α* and *HIF2α* in both primary pterygium (11% and 38%) and recurrences (18% and 21%) when compared to healthy conjunctival tissue (46% and 66%). Differences between immunoreactivity of *HIF1α* and of *HIF2α* in primary pterygium and controls were each highly significant (*p* < .001). Within the group of primary pterygium, epithelial immunoreactivity of *HIF2α* (38%) was significantly higher than that of *HIF1α* (11%). In recurrent pterygium and healthy conjunctiva, immunoreactivity levels of *HIF2α* were higher than those of *HIF1α* as well; however, differences between both isoforms were not significant.

**Conclusion:**

Our study shows evidence that the higher expressed epithelial *HIF2α,* rather than *HIF1α*, and the balance between both HIF isoforms might be relevant factors associated with pathogenesis of primary pterygium. Modulation of *HIF2α* levels and activity may thus offer a new therapeutic approach to the treatment of advancing pterygium where the initial stage with its HIF1-peak has already passed.

## Introduction

The pathogenesis of pterygium, a wing-like shaped ocular surface disorder possibly affecting eyesight and cosmetic appearance, is considered to be related to limbal stem cell deficiency mainly of the nasal cornea predominantly due to chronic ultraviolet irradiation reflecting through the whole cornea from the temporal side [1]. Conjunctival pterygium is associated with inflammation and distinctive neovascularization, and increased expression of the vascular endothelial growth factor (VEGF) [2]. As a consequence, vascular density is enhanced in pterygium [3]. Interestingly, at microscopic level, not the increased blood vessel density, but an enhanced lymph vessel density is associated with a shorter recurrence time [4]. As both angiogenesis and lymphangiogenesis are mediated by the hypoxia-inducible transcription factors [5], it is plausible to assume that these key factors for vessel formation might be involved in pterygium development.

Hypoxia-inducible factors (HIFs) are transcription factors of the basic helix-loop-helix (bHLH)/PAS family that perceive oxygen availability and are known to play a decisive role in adaptive cellular responses to hypoxia and inflammation. Under hypoxic as well as under non-hypoxic HIF-promoting conditions such as inflammation, stabilized HIF migrates from the cytoplasm into the nucleus [6].

The HIF family includes *HIF1α*, which is expressed in most tissues and regulates the majority of HIF target genes, *HIF2α*, showing a more restricted tissue expression with exclusive and cell-type-dependent target genes, and HIF3α, which seems to be a *HIF1α* target gene possibly functioning as a modulator of hypoxic gene induction [7, 8].

It could be demonstrated that *epithelial* cells show a significantly higher activation of HIF target genes than mesenchymal cells [9]. HIFs lead to diverse tissue responses through gene expression inducing angiogenesis and metabolic reprogramming, e.g., via Erythropoietin (EPO) or VEGF, as well as proinflammatory responses [6, 8].

Furthermore, HIFs are involved in tumor formation and its progression [8]. Regarding the ocular adnexa, an increased *HIF1α* rate in malignant tumors such as squamous cell carcinomas is associated with an unfavorable clinical outcome [10].

Until today, only the *HIF1α* protein has been studied in pterygium [11]. Pagoulatos et al. found a statistically significant increased expression of *HIF1α* and of heat shock proteins Hsp27, Hsp70, and Hsp90 in pterygium compared to healthy conjunctiva [11]. Similar results were recently presented by Dong et al. regarding *HIF1α* and STAT3, a DNA binding protein that regulates various biological processes [12]. These findings were interpreted as an adaptive process for cell survival under stressful conditions such as UV irradiation or hypoxia. Pagoulatos et al. suggest that the activation of *HIF1α* in pterygium may not only be the result of hypoxia, but also the result of hypoxia independent mechanisms, such as oncogene activation and growth factor signal pathway. This is plausible for pterygium being a lesion of superficial cornea and conjunctival tissue which is always exposed to environmental oxygen unless when keeping the eyes closed.

Besides *HIF1α,* no other HIF-isoform, e.g., *HIF2α,* has been described in pterygium up to now.

The aim of this study using immunohistochemistry (IHC) was to investigate whether in pterygium besides *HIF1α*, also *HIF2α* is expressed. And if so, the ratio between both isoforms should be evaluated in primary pterygium, recurrent pterygium and healthy conjunctiva*,* as possible pathogenetic factors.

## Materials and methods

### Patient and tissue data

A total of 125 patients who underwent pterygium surgery at the Eye Centre, Medical Centre—University of Freiburg, between 2006 and 2013 (47 females, 78 males, mean age 60 years, age range 17–87 years) were included in this study (Table 1). A total of 98 patients were diagnosed with primary pterygium (104 eyes), and 28 patients had recurrent pterygium (28 eyes) with 1 patient belonging to both groups. Pterygium removal was performed under retrobulbar anesthesia.

**Table 1:** Numbers of patients and specimens included in this study

Groups	Number of patients	Number of eyes	Prior surgery	*HIF1α*-analysis (number of eyes)	*HIF2α*-analysis (number of eyes)
Primary pterygium	98	104	none	55	84
Recurrent pterygium	28	28	single pterygium excision	6	28
Control group	20	20	none	20	20

In the control group, an excess of healthy bulbar conjunctiva was excised in 20 patients receiving retinal buckling procedures for retinal detachment between 2013 and 2015 (7 females, 13 males; mean age 64 years, range 43–86 years). None of the patients had previous ocular surgery.

### Paraffin embedding

Formalin fixation and paraffin embedding of pterygium and healthy conjunctiva specimens were performed immediately after surgery according to our routine protocols, as previously described [13]. Briefly, specimens were fixed in 4% formalin for 12 h, dehydrated in alcohol, and finally processed for paraffin embedding. 4 μm-thick sections were cut, mounted on silanized slides and deparaffinized in xylol-alcohol. Following routine histological staining, each specimen’s histological diagnosis was provided by an experienced ophthalmic pathologist (CAH).

### IHC staining and evaluation of results

Two serial histological slides from each specimen were separately stained for *HIF1α* and *HIF2α,* respectively, using the Catalyzed Signal Amplification (CSA) System II (Code K1497) (Dako Corporation, Carpinteria, CA), a highly sensitive immunohistochemical staining procedure. The method is based on peroxidase-catalyzed deposition of a fluorescein-labelled phenolic compound, followed by a secondary reaction with a peroxidase-conjugated anti-fluorescein [14]. Briefly, slides were deparaffinized, rehydrated in graded alcohols, and placed in Tris-buffered saline solution. Antigen retrieval was performed by autoclaving the slides for 20 min in citrate buffer (pH 6.0). Endogenous peroxidase was blocked by incubating the slides in 0.3% H2O2 for 15 min. Sections were incubated for 15 min (anti-*HIF1α*) and 60 min (anti-*HIF2α*) at room temperature followed by an overnight incubation with the primary antibodies anti-*HIF1α* (clone H1alpha67 Novus Biologicals, Inc., Littleton, CO; 1:8000 dilution) and anti-*HIF2α* (clone 190b; Santa Cruz Biotechnology, Inc., Dallas, TX; 1:500 dilution) at 4 °C. Secondary antibody was applied for 60 min. Sections were incubated for 15 min with an amplification reagent. Anti-fluorescein solution was applied, and the sections were incubated for 15 min. Finally, peroxidase activity upon AEC (3-amino-9-ethylcarbazole) solution led to the formation of a red-brown reaction product. In between these steps, slides were washed in Tris-buffered saline. Harris’ hematoxylin was used to counterstain the slides. A positive and a negative control slide were included in each series.

Specimens of 55 eyes with primary pterygium, 6 eyes with recurrent pterygium (all 61 cases with follow-up data) and 20 control specimens were stained with antibodies against *HIF1α*. Meanwhile, specimens from additional eyes had accumulated in our archive, so that in a second experimental series a total 84 eyes with primary pterygium (35 cases with follow-up data), 28 eyes with recurrent pterygium (6 cases with follow-up data) and 20 control cases were available for staining with antibodies against *HIF2α*, which has not been described in the literature to date for pterygium (Table 1).

Representative areas of each section of conjunctival epithelium were selected. Photographs of nuclear granules were taken on all focus levels and then superimposed into one photograph using the “Fusion Free” software [15]. These superimposed photographs were analyzed at 400 × magnification for HIF positivity, manually marked and automatically counted using an in-house planimetry tool based on open-source software components; data were aggregated in R (Version 3.3.2).

All positively stained cells were counted and their percentage of all counted epithelial cells was compared between the groups of primary and recurrent pterygium as well as control specimens. Expectedly, HIF positivity was mainly nuclear [6] and only cells with nuclear positivity were taken into account since the nuclear form is mainly relevant for subsequent gene activation of HIF-dependent factors [8]. Faint cytoplasmic staining was noted, including in the goblet cells, which cannot completely be ruled out as being background staining. Occasional positive nuclear staining of goblet cells was not included in our analysis.

### Assessment of pterygium vascular density in slit lamp photographs of HIF1-stained cases with follow-up

Color images were converted to grayscale using a green filter and a high-pass filter and the contrast was preprocessed followed by an h-dome operation (http://www.leptonica.com/grayscale-morphology.html) and further contrast enhancement. This procedure resulted in a color change of the red vessels into white on a black background. The image was then thresholded and segmented into vessels and background. Finally, the ratio of pixels that were identified as vessels was calculated in relation to the total number of pixels [3, 16, 17]. The region of interest was manually defined and vascular density was automatically analyzed using an "Open-source" planimetry and image analyzing platform (https://github.com/daboe01/Cellfinder) which is based upon the Open-Source Projekts ImageMagick (www.imagemagick.org), the programming language "R" (https://www.r- project.org/) with the image analyzing module EBImage (https://bioconductor.org/packages/release/bioc/html/EBImage.html) and the programming library Leptonica (http://www.leptonica.com/).

### Immunoblotting

Two pterygia and two conjunctival specimens were used for testing the *HIF2α* antibody by immunoblotting using standard procedures. *HIF1α* has already been validated by Pagoulatos et al. [18]. Specimens were put in 100 µl of lysis buffer (150 mM NaCl, 1% Triton X-100, 20 mM HEPES pH 7.4, protease inhibitor mix (MSSAVE, Sigma, Taufkirchen, Germany)) on ice immediately after surgery to prevent degradation and homogenized with a hand homogenizer. 75 µg of protein as determined by BCA assay were applied to a polyacrylamide gradient gel, blotted to a PVDF membrane, and blocked in TBST (10 mM Tris, 150 mM NaCl, 0,1% Tween-20, pH 7,4) containing 3% BSA. Antibody *HIF2α* (clone 190b, Santa Cruz Biotechnology, Inc., Dallas, TX) was applied at a dilution of 1:1000 overnight. After washing, a secondary antibody (1:10,000, goat-anti-mouse-HRP, 115-035-003, Jackson Immunoresearch Europe, Ely, UK) was applied for 2 h, and after washing again, an anti-GAPDH antibody labelled with DL680 (1:2000, MCA4739D680, Biorad, München, Germany) was applied for 1 h. After washing, the blot was imaged with ECL (Amersham RPN2109, GE Healthcare, München, Germany).

### Statistical analysis

Analysis of variance (ANOVA) between groups was performed in Microsoft Excel (2016) with XLSTAT Base (2018) [19] software add-in using Tamhane’s T2 test, an all-pairs comparison test for normally distributed data with unequal sample size and unequal variances. Bar graphs were created with Microsoft Excel (2016).

## Results

### HIF immunoreactivity

**Fig. 1:**
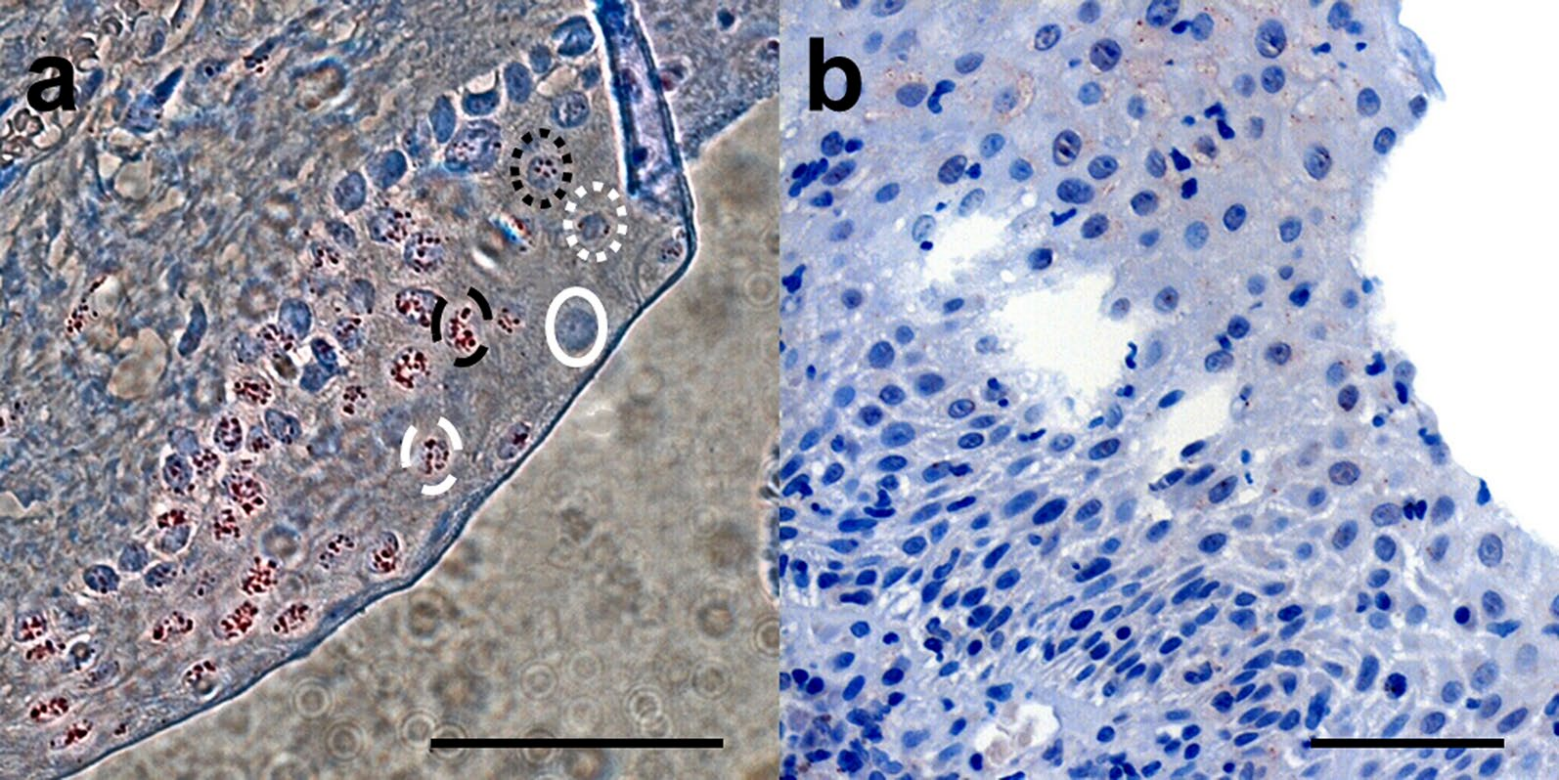
Immunoreactivity patterns. **a** Exemplary histological microscope image of a primary pterygium immunohistochemically stained against *HIF1α* after superimposing all focus levels of the selected area with the “Fusion Free” software. Identified patterns of *HIF1α* stained granules within the nuclei of epithelial cells: 1 (circle with small white dots)—few small granules, 2 (circle with small black dots)—numerous small granules, 3 (lined white circle)—few large granules, 4 (lined black circle)—numerous large granules, 5 (white circle)—no granules (= negative); bar = 100 µm **b** Negative control (pterygium without primary antibody, conventional microscopy); bar = 100 µm

*HIF1α* and *HIF2α* immunoreactivity was present in primary and recurrent pterygium. Figure 1 a shows an exemplary histological microscope image of a primary pterygium after superimposing all focus levels of the selected area with the “Fusion Free” software. Within the epithelium, 5 different patterns of HIFα stained granules were identified: *pattern 1*—few small granules, *pattern 2*—numerous small granules, *pattern 3*—few large granules, *pattern 4*—numerous large granules, *pattern 5*—no granules (= negative). Granules with a diameter of 1.2–1.6 µm were categorized as “small” and those with a diameter of > 1.6–2.2 µm were categorized as “large”. In Fig. 1b, a representative negative control is shown.

Cells exhibiting these patterns were counted and assigned to 4 categories (Table 2).

**Table 2:** Categories and assigned patterns of HIFα stained granules (Fig. 1a)

Category	Description
1	Negatively stained nuclei (*pattern 5*) and nuclei with few small^a^ granules (*pattern 1*)
2	Nuclei with numerous small^a^ granules (*pattern 2*)
3	Nuclei with few large^b^ granules (*pattern 3*)
4	Nuclei with numerous large^b^ granules (*pattern 4*)

^a^Small: 1.2–1.6 µm diameter

^b^Large: > 1.6–2.2 µm diameter

**Fig. 2:**
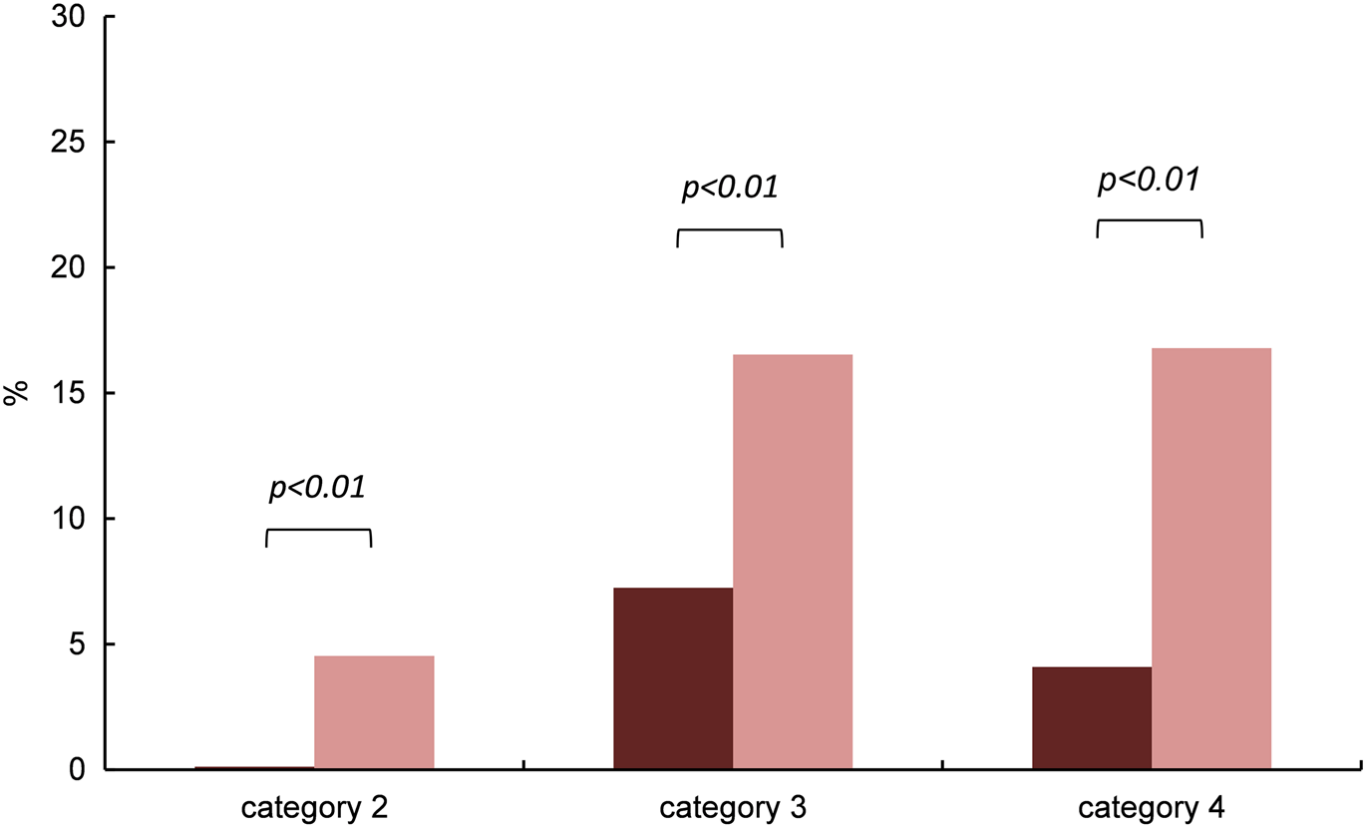
Categories of epithelial immunoreactivity. Epithelial immunoreactivity of *HIF1α* (darker columns, left, *n* = 55) and *HIF2α* (brighter columns, right, *n* = 84) in primary pterygium (PP) within categories 2, 3 and 4 (columns represent mean relative immunoreactivity). *α* = 5%, *p* < 0.01

Analysis of variance (ANOVA) of epithelial *HIF1α* and *HIF2α* immunoreactivity performed within each category 2, 3 and 4 in pterygium, recurrences and controls revealed significant differences between *HIF1α* and *HIF2α* in primary pterygium. Immunoreactivity of *HIF2α* was found to be significantly higher than that of *HIF1α* in each of categories 2, 3 and 4. Results are given as relative mean (% of all counted epithelial cells within each specimen) with the respective standard deviation. In category 2, HIF1 α was 0.12% ± 0.60% and *HIF2α* was 4.5% ± 11% (*p* = 0.0015); in category 3, HIF1 α was 7.2% ± 13% and *HIF2α* was 17% ± 17% (*p* = 0.0012); in category 4, HIF1 α was 4.1% ± 13% and *HIF2α* was 17% ± 26% (*p* = 0.0011) (Fig. 2).

For further calculations, nuclei in category 1 were classified “negatively stained”, and nuclei in categories 2–4 summarized and defined as “positively stained” for *HIF1α* and *HIF2α*, respectively. In the epithelium of primary pterygium, 28 of 55 specimens (51%) stained positive for *HIF1α*, and 73 of 84 specimens (87%) for *HIF2α*. In the epithelium of recurrent pterygium, 3 of 6 specimens (50%) stained positive for *HIF1α*, and 19 of 28 specimens (68%) for *HIF2α*. In healthy conjunctiva, 20 of 20 specimens (100%) stained positive for both *HIF1α* and *HIF2α*. For a conservative approach, all specimens of each group were included in the following calculations of the mean immunoreactivity of *HIF1α* and *HIF2α*.

**Fig. 3:**
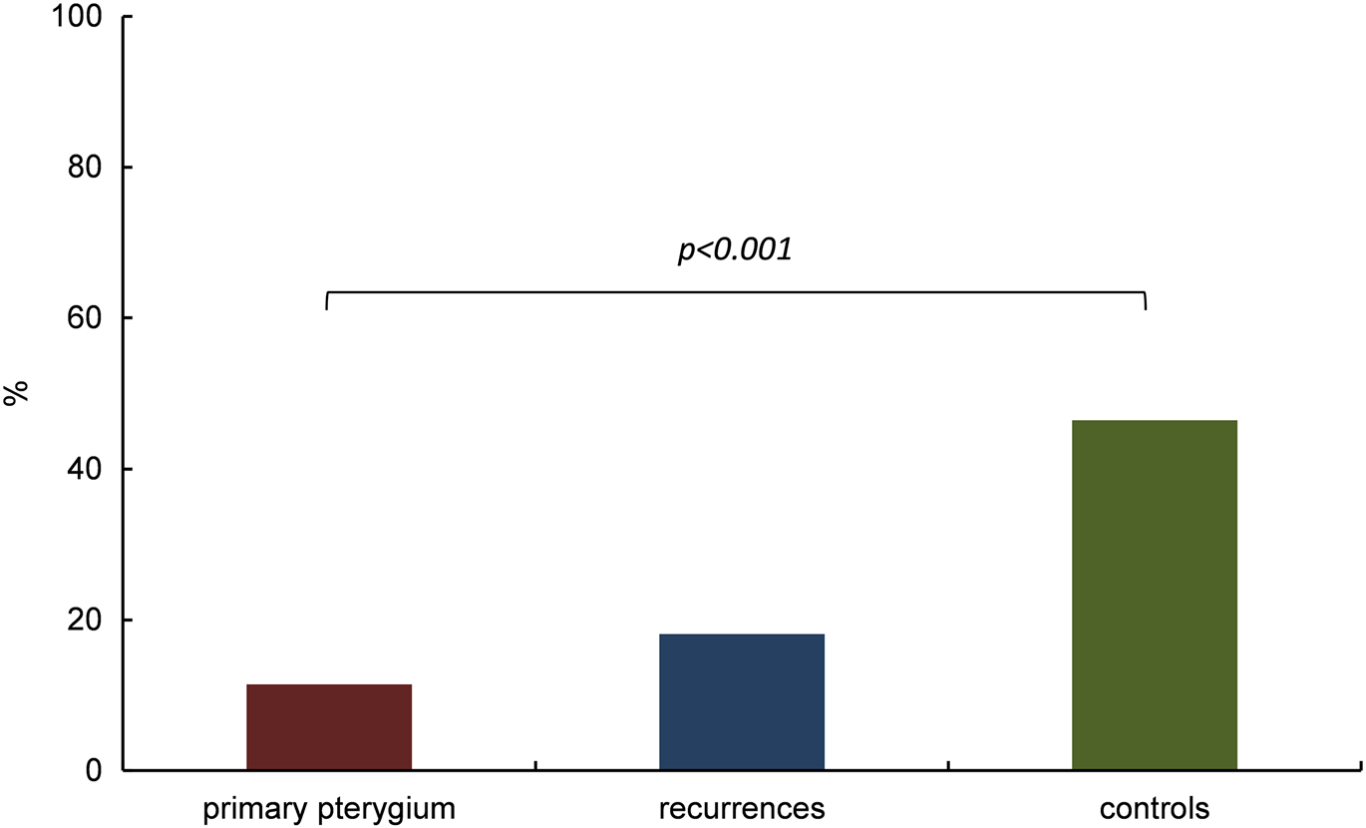
Immunoreactivity of *HIF1α* in pterygium and controls. Comparison of epithelial *HIF1α* in primary pterygium (*n* = 55), recurrences (*n* = 6) and controls (*n* = 20); columns represent mean relative immunoreactivity. Epithelial *HIF1α* immunoreactivity was statistically significantly higher in healthy conjunctival tissue (46%, controls, right column) than in primary pterygium (11%, left column), *α* = 5%, *p* < 0.001, while immunoreactivity in the recurrences was slightly yet not significantly higher (18%, mid column)

**Fig. 4:**
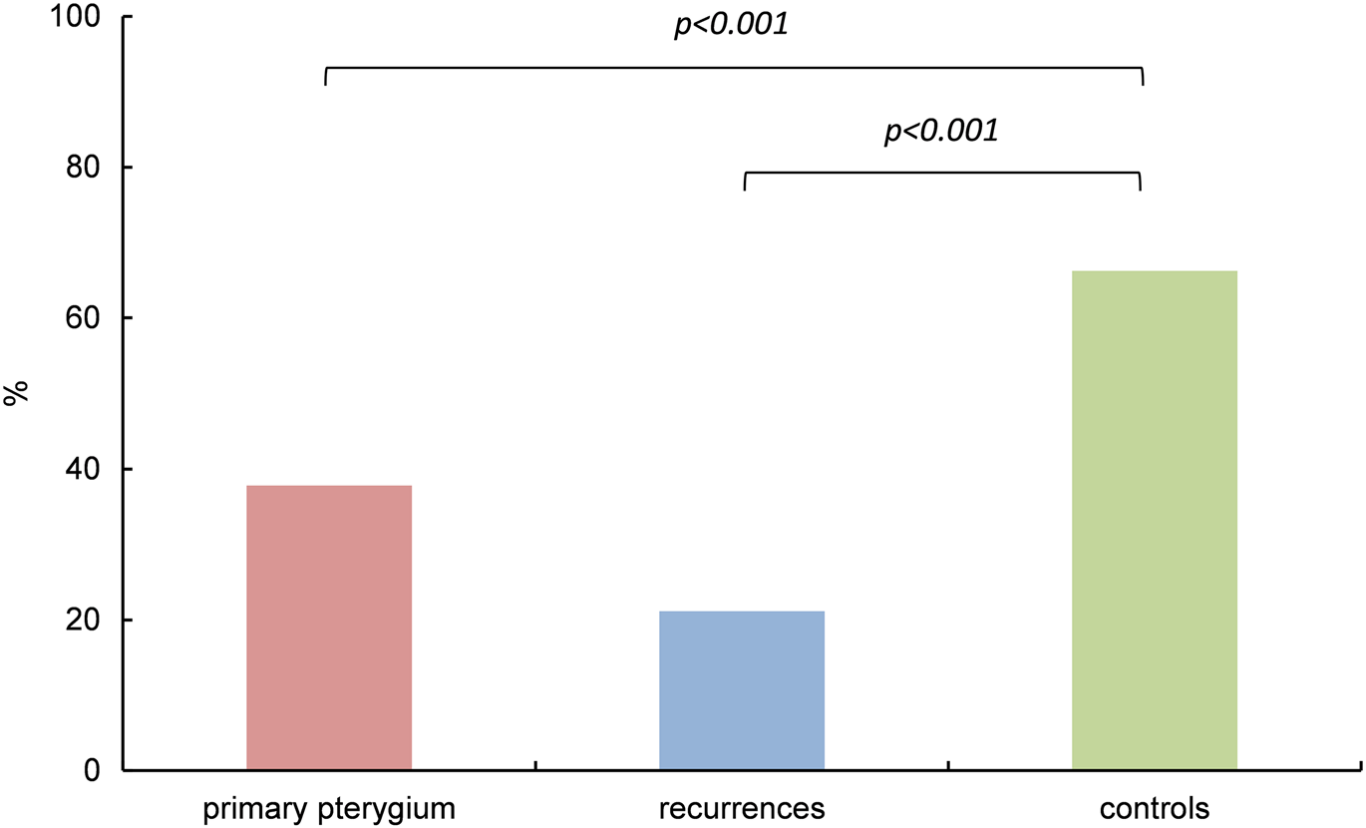
Immunoreactivity of *HIF2α* in pterygium and controls. Comparison of epithelial *HIF2α* in primary pterygium (*n* = 84), recurrences (*n* = 28) and controls (*n* = 20); columns represent mean relative immunoreactivity. *HIF2α* immunoreactivity was statistically significantly higher in healthy conjunctival tissue (66%, controls, right column) than in primary pterygium (38%, left column) as well as in recurrent pterygium (21%, mid column). *α* = 5%, *p* < 0.001

Results are given as relative mean (% of all counted cells within each specimen) with the respective standard deviation. Unexpectedly, primary and recurrent pterygium revealed lower immunoreactivity of *HIF1α* (11% ± 20%, and 18% ± 36%) and *HIF2α* (38% ± 31%, and 21% ± 27%), respectively, in epithelial cells when compared to healthy conjunctiva (*HIF1α*: 46% ± 30% and *HIF2α*: 66% ± 31%). The observed difference of epithelial *HIF1α* and *HIF2α* immunoreactivity in primary pterygium vs healthy conjunctival tissue was statistically significant (*p* < 0.001 and *p* = 0.003, respectively) (Figs. 3, 4), as was the difference of epithelial *HIF2α* immunoreactivity in recurrent pterygium vs that in healthy conjunctiva (*p* < 0.001) (Fig. 4).

**Fig. 5:**
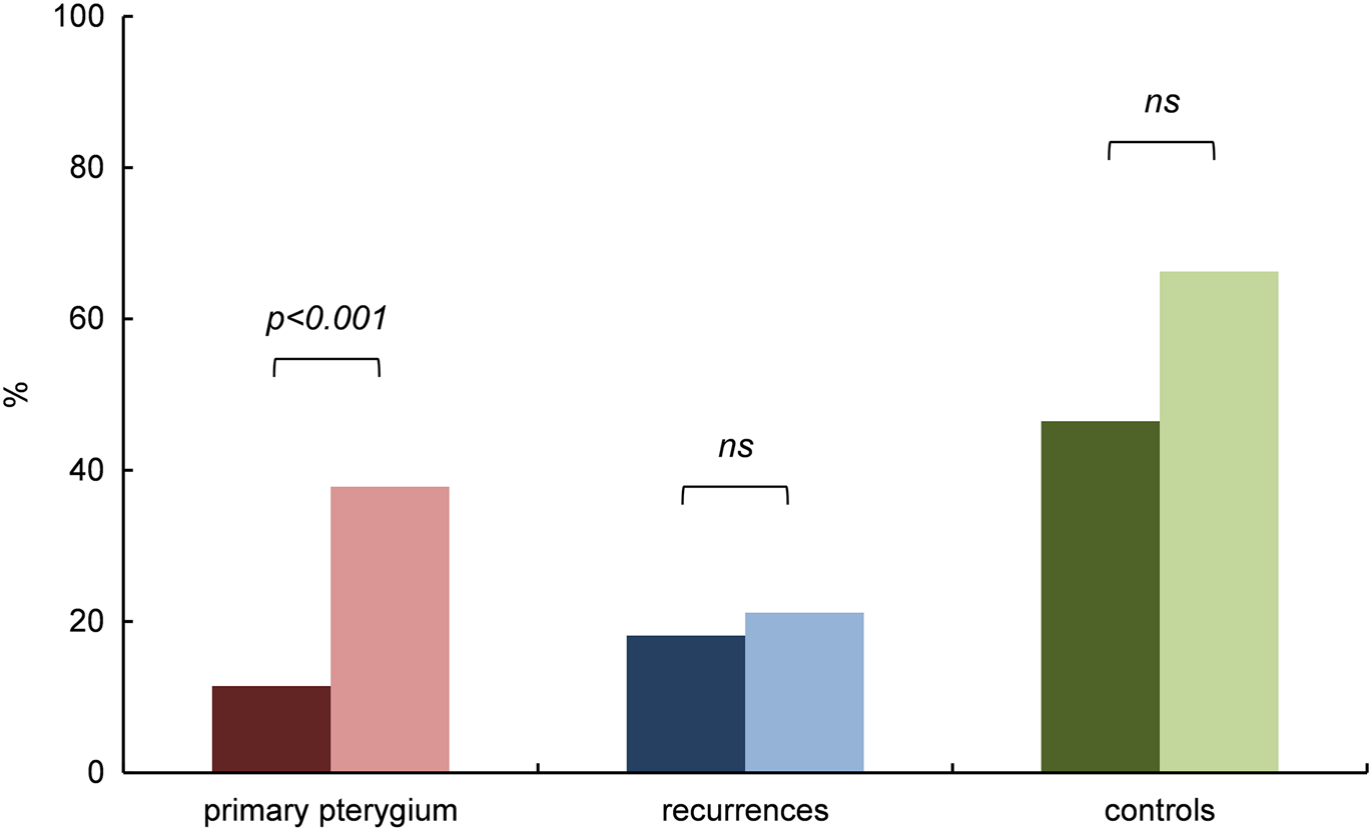
Immunoreactivity of *HIF1α* and *HIF2α* in pterygium and controls. Comparison of epithelial *HIF1α* (darker columns, left) and *HIF2α* (brighter columns, right) in primary pterygium (*n* = 55, and *n* = 84), recurrences (*n* = 6, and *n* = 28) and controls (*n* = 20, and *n* = 20); columns represent mean relative immunoreactivity. Within the group of primary pterygium, *HIF2α* immunoreactivity (38%) was significantly higher than that of *HIF1α* (11%), *α* = 5%, *p* < 0.001. In both other groups, differences between epithelial *HIF1α* and *HIF2α* were not significant (ns), however, the *p*-value for comparisons in the controls was *p* = 0.05

Within the group of primary pterygium, the epithelial immunoreactivity of *HIF2α* was significantly higher than that of *HIF1α* (*p* < 0.001). In both other groups, we also found higher immunoreactivity of *HIF2α vs HIF1α* (Fig. 5); these differences were not significant, however, we obtained *p* = 0.05 for comparisons in the controls. There was no correlation between the levels of *HIF1α* and *HIF2α* immunoreactivity *within* identical primary pterygium specimens.

**Fig. 6:**
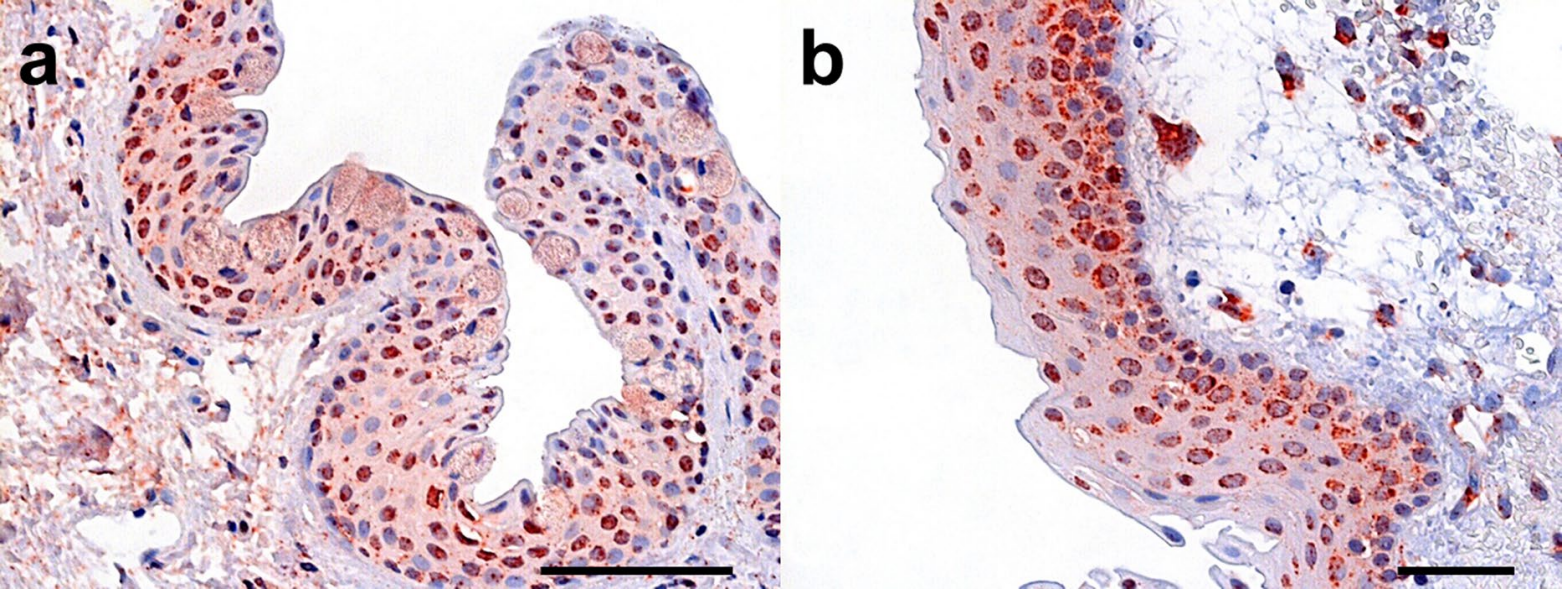
Immunohistochemical stainings of normal conjunctiva. Representative immunohistochemical stainings of normal conjunctiva for *HIF1α* (**a** bar = 200 µm) and *HIF2α* (**b** bar = 100 µm) showing strong nuclear positivity. Faint cytoplasmic staining was noted, including in the goblet cells, which cannot be completely ruled out as being background staining

**Fig. 7:**
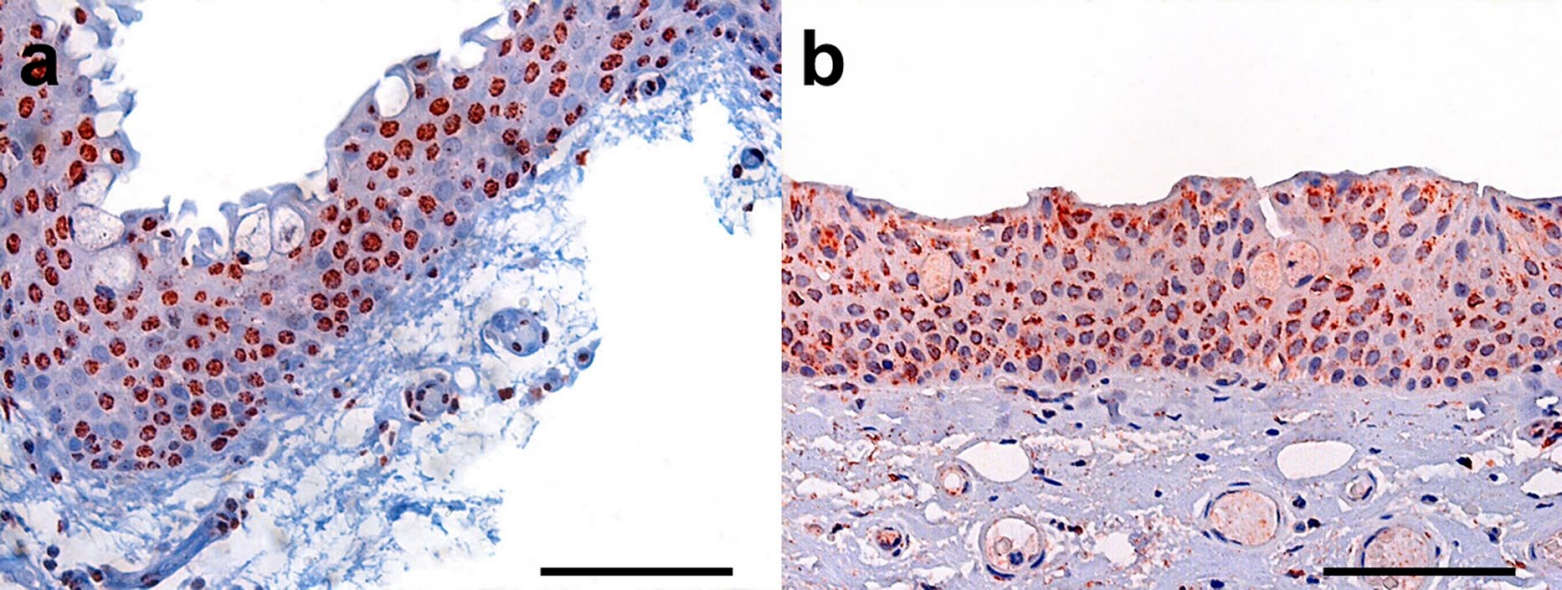
Immunohistochemical stainings of primary pterygium. Representative immunohistochemical stainings of primary pterygium for *HIF1α* (**a**, bar = 200 µm) and *HIF2α* (**b**, bar = 200 µm) showing strong nuclear and slight cytoplasmic positivity, which also cannot be completely ruled out as being background staining

Representative immunohistochemical stainings of normal conjunctiva and primary pterygium for *HIF1α* and *HIF2α* are shown in Figs. 6 and 7.

### *HIF2α* immunoblotting

**Fig. 8:**
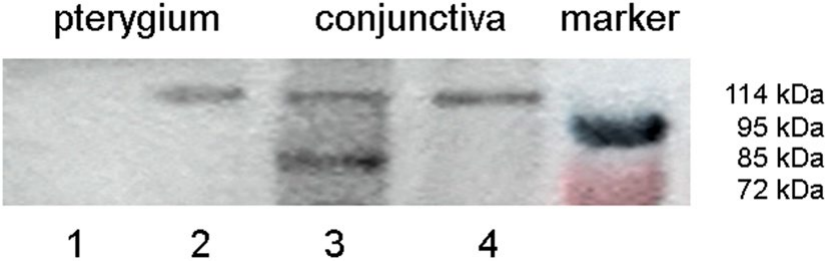
*HIF2α* immunoblot analysis. Immunoblotting experiments on tissue extracts from two pterygium specimens (2 left lanes: #1 and #2) and two normal conjunctival samples (2 right lanes: #3 and #4) were performed to confirm the expression of *HIF2α* as detected with immunohistochemistry. These experiments demonstrate the presence of the expected 114-kD band of the *HIF2α* protein in one pterygium and both normal conjunctivae (lanes #2-#4). One additional band at 85 kD represents a post-translational modification of *HIF2α*. Right lane with molecular mass markers: red 72 kD, black 95 kD

Immunoblot analysis of tissue extracts from two pterygium and two normal conjunctiva samples is shown in Fig. 8 where the extracts were probed with anti-*HIF2α*. The same amount of protein (75 μg) was loaded into each lane. Positions of molecular mass markers (red 72 kD, blue/black 95 kD) are indicated on the right. The *HIF2α* exhibited a specific band at 114 kD and was detected in one pterygium and both control conjunctivae. One additional band at 85 kD represents a post-translational modification of *HIF2α*.

### Vascular density

In 32 primary pterygia, vascular density ranged from 15 to 39% of the analyzed area (median/mean 24%). No correlation to the levels of *HIF1α*- and *HIF2α*-immunoreactivity was observed (data not shown).

## Discussion/conclusion

Pterygium represents an ocular surface lesion, which is characterized by epithelial and fibrovascular invasions toward the central portion of the cornea. The family of HIF transcription factors and their target genes mediates the response to non-hypoxic HIF-promoting conditions, e.g., inflammation [6] and may play an important role in the pterygium lesion’s pathogenesis and growth. In this study, we investigated the immunoreactivity patterns of *HIF1α* and *HIF2α* isoforms in primary and recurrent pterygium and in healthy conjunctiva (control specimens), using immunohistochemistry.

### *HIF1α* and *HIF2α* immunoreactivity in pterygium vs controls

Aspiotis et al. reported significantly higher expression levels of VEGF, one of the main HIF target genes, in 46% of 52 cases of pterygium (51 primary, 1 recurrent) in stromal and vascular endothelial cells, compared to those in 7 healthy conjunctiva [2]. Epithelial pterygium cells showed significantly lower VEGF immunoreactivity than stromal cells. Immunoreactivity of Thrombospondin-1 (THBS1), which may inhibit angiogenesis [20], was low in pterygium so that inducers of angiogenesis are more likely to act uninhibited [2]. Since VEGF expression is known to be enhanced and THBS1 expression repressed by HIFα [21], analysis of *HIF1α* and *HIF2α* expression in pterygium seemed promising to increase our understanding of the pathogenesis of primary and recurrent pterygium.

Our immunohistochemical results first show that both *HIF1α* and *HIF2α* are expressed in the epithelium of 51% and 87%, respectively, primary pterygium specimens and in the epithelium of 50% and 68%, respectively, recurrent pterygium specimens. Surprisingly, we detected significantly lower epithelial immunoreactivity of both HIF isoforms in primary pterygium compared to that in healthy conjunctiva (Figs. 3 and 4). Similar to our results, Aspiotis et al. found a higher VEGF expression level in *epithelial* cells of healthy conjunctiva (83%) obtained during cataract surgery, compared to pterygium (58%) [2].

However, our findings are in contrast to Pagoulatos et al.’s and Dong et al.’s findings for *HIF1α* [11, 12], who report significantly increased immunoreactivity of *HIF1α* in pterygium compared to healthy conjunctiva. However, their control specimens originated from glaucoma or cataract surgery, and from strabism or ocular trauma surgery, respectively, with no information on when the tissue was removed during surgery. While excision of pterygium is performed within less than 5 min, our control specimens were excised at the end of retinal buckling surgery, which usually lasts 30–45 min. The associated conjunctival trauma may have sufficed to considerably upregulate HIF immunoreactivity within the tissue, especially in the most distal conjunctival segment removed. For instance, traumatic brain injury in rats resulted in significant *HIF1α* elevation detected as early as 1 h after the trauma [22]. We had found similar results when immunohistochemically comparing immunoreactivity of both HIF isoforms in conjunctival intraepithelial neoplasia (CIN) and in healthy conjunctiva, and assumed that HIF is downregulated in CIN to avoid cell cycle arrest and HIF-induced apoptosis, because all cells of the conjunctiva, even if dysplastic, may be well oxygenated [13].

Neither Pagoulatos et al., Aspiotis et al. nor Dong et al., provide surgery details [2, 11, 12] that would clarify in particular whether the healthy conjunctiva were excised at the beginning or at the end of the surgery, which might have otherwise helped to explain the observed level of HIF or VEGF immunoreactivity. In retrospect, the risk of higher HIF immunoreactivity in our control samples due to the longer time span until excision may thus be considered a limitation of their suitability; however, no alternative control material was available. For further studies investigating HIF and its downstream factors in conjunctival specimens, excision details for healthy conjunctiva used as control material will be of importance to enable accurate interpretation of HIF expression with confidence.

Another aspect that might be responsible for our findings is the fact that we evaluated *nuclear* HIF positivity, which we consider to reflect actual HIF activity, while *cytoplasmic HIF1α* was analyzed by Dong et al. [12].

We would like to make yet another point that may also contribute to epithelial *HIF1α* and *HIF2α* being less upregulated than expected in our primary pterygium.

Our literature search showed that ocular hypoxia was so far not identified as an initial pathogenetic factor in development of pterygium. Several authors found increased vascular density in advanced pterygium, even in the epithelium [23], rather indicating norm- or even hyperoxia [2, 24]. We hypothesize that chronic non-hypoxic HIF-promoting conditions, e.g., inflammation with subsequent localized hypoxia during development and progression of pterygium may result in higher epithelial HIF-levels compared to “inactive” or stationary pterygium with capillaries occasionally grown into the epithelium and rather normoxic conditions [23], under which *HIF1α* is kept at a low level by degradation via the 26S-proteasome [25]. Further, the progressive (active) pterygium is known to have the greater potential for recurrence [26, 27]. We found only 4 recurrences of the 55 primary pterygium included in this study within 24–32 months postoperatively, indicating that our cohort may comprise mainly “inactive” pterygium with rather normoxic conditions and consequently with lower HIF-levels. Due to the statistically insufficient low number of recurrences, however, we refrained from comparing epithelial HIF immunoreactivity in the 4 primary pterygium cases with recurrences with those without recurrences. In conclusion, we postulate that the relative accumulation of *HIF1α* and *HIF2α* in epithelial tissue of our control conjunctiva may reflect its production under traumatic intraoperative condition, while its lower levels in pterygium reflect the low angiogenic activity in advanced, well vascularized pterygium stage.

### *HIF1α* vs *HIF2α* immunoreactivity in pterygium and controls

Significantly higher immunoreactivity levels of *HIF2α* compared to *HIF1α* were found in primary pterygium by immunohistochemistry analysis (*p* < 0.001; *HIF2α*:*HIF1α* = 3.5). Furthermore, epithelial immunoreactivity of *HIF2α* was significantly increased compared to *HIF1α* also within each individual category 2 (*HIF2α*:*HIF1α* = 38), 3 (*HIF2α*:*HIF1α* = 2.4) and 4 (*HIF2α*:*HIF1α* = 4.1) with assigned patterns of *HIFα* stained granules (2: numerous small granules, 3: few large granules, 4: numerous large granules).

Similar but weaker differences were immunohistochemically seen in epithelia of recurrent pterygium (not significant; *HIF2α*:*HIF1α* = 1.2) and of control specimens (*p* = 0.05; *HIF2α*:*HIF1α* = 1.4). When interpreting the result, it should be considered that the affinity of the antibody to the specific antigen may vary. However, the difference between the immunoreactivity of *HIF1ɑ* and *HIF2ɑ* in primary pterygium is quite high (ratio *HIF2ɑ*/*HIF1ɑ* > 30 in small granules) with significantly smaller ratio in the control specimens. We therefore consider this finding a clear indication of an actually increased *HIF2ɑ* production compared to that of *HIF1ɑ*. The small number of recurrent pterygium specimens (*n* = 6) is disproportionally small for robust statistics and must therefore be interpreted with caution. Befani and Liakos [28] consider *HIF2α,* which upregulates several genes involved in almost every step of angiogenesis, at least as important for the regulation of physiological and pathophysiological angiogenesis as *HIF1α*. Under hypoxic conditions, the main angiogenic factor VEGF is induced directly by *HIF2α* having a stronger transactivation activity on its promoter than *HIF1α* that also regulates VEGF [29].

Dengler et al. describe upregulation of *HIF2α* in periods of *chronic* hypoxia, whereas *HIF1α* predominates under conditions of *acute* hypoxia stimulating glycolytic genes [8, 30]. In other words, *HIF1α* drives the initial response to hypoxia but during chronic hypoxic exposure, *HIF2α* takes over and drives the chronic response [31]. These aspects seem plausible also for pterygium being a chronic transformation of the conjunctiva with neovascularization and possible stem cell degradation. As previously mentioned, in pterygium, non-hypoxic HIF-promoting factors like inflammation rather than hypoxia seem to play a role.

Taylor et al. investigated the nuclear distribution of both isoforms *HIF1α* and *HIF2α* in the nuclei of HeLa cells by fluorescent microscopy. While upon exposure to hypoxia *HIF1α* accumulates homogeneously in the nucleus, *HIF2α* is localized in the nucleus in speckles near the active polymerase RNA, providing easier access to the promoters of target genes [32]. The authors conclude that concentration of *HIF2α* into speckles may explain its increased stability and protection from degradation compared with *HIF1α*. Moreover, the storage of active transcription factors near to active transcription sites thus enhancing their availability and activity enables rapid changes in gene expression, which is crucial in the response to environmental stress such as oxygen deprivation [32].

Furthermore, it is widely accepted that HIF expression is not only induced by hypoxia but also by other forms of pathological stress such as inflammation [6], which has been described for various mammalian cells, including epithelial and endothelial cells [33, 34]. Inflammatory conditions lead to an increase in intracellular oxygen utilization and local tissue hypoxia, which activates the HIF signalling pathway [35]. For instance, differential expression of both HIF isoforms was seen in inflammatory joint conditions; immunostaining of synovial membrane specimen from patients with rheumatoid arthritis, composed entirely of connective tissue, showed that *HIF2α* (> 60%), but not *HIF1α* (< 10%) was highly expressed [36]. In the lung, the predominant isoform was found to vary over time during an inflammatory response, whereby *HIF2α* seems to predominate in the latter, reparative stages of inflammation [37].

Increased immunoreactivity of *HIF2α* versus *HIF1α* in the pterygium included in our study might therefore be associated with chronic inflammation frequently coexisting with (localized) hypoxia, and histologically diagnosed in most of our pterygium specimens.

We conclude that epithelial *HIF2α,* rather than *HIF1α*, and the balance between both HIF isoforms play an essential role in the complex transcriptional regulation of the cell response and adaptation to pathological stress conditions such as initial chronic epithelial inflammation (rather than chronic hypoxia), and thus in the pathogenesis and growth of primary pterygium. Modulation of *HIF2α* levels and activity may thus offer a new therapeutic approach to the treatment of advancing pterygium, where the initial stage with its *HIF1ɑ*-peak has already passed. PT2399, an *HIF2α*-antagonist which seems to be effective upon renal cell carcinoma in human [38] and pancreatic adenocarcinoma in mice [39], could be a therapeutic option as subconjunctival injection or eye drops in advancing primary pterygium, restoring normal *HIF2α/ HIF1α* balance. This might not be effective in recurrence pterygia with much lower *HIF2α/ HIF1α* ratio. Further studies are required for a more detailed understanding of the role of *HIF2α* in molecular regulatory pathways and its relevance as a therapeutic target in pterygium.
